# Removal of SDS from biological protein digests for proteomic analysis by mass spectrometry

**DOI:** 10.1186/s12953-016-0098-5

**Published:** 2016-09-06

**Authors:** Soundharrajan Ilavenil, Naif Abdullah Al-Dhabi, Srisesharam Srigopalram, Young Ock Kim, Paul Agastian, Rajasekhar Baaru, Ki Choon Choi, Mariadhas Valan Arasu, Chun Geon Park, Kyung Hun Park

**Affiliations:** 1 Grassland and Forage Division, National Institute of Animal Science, RDA, Seonghwan-Eup, Cheonan-Si, Chungnam, 330801 Korea; 2 Department of Botany and Microbiology, Addiriyah Chair for Environmental Studies, College of Science, King Saud University, P. O. Box 2455, Riyadh, 11451 Saudi Arabia; 3 Department of Medicinal Crop Research, Rural Development Administration, Eumseong, Chungbuk, 369-873 Republic of Korea; 4 Research Department of Plant Biology and Biotechnology, Loyola College, Nungambakkam, Chennai-34, Tamil Nadu India; 5 Labmate (Asia) Pvt. Ltd, Chennai, Tamil Nadu 600015 India

**Keywords:** MOFs, SDS removal, Biological sample, Proteomic analysis

## Abstract

**Background:**

Metal-organic frameworks (MOFs - MIL-101) are the most exciting, high profiled developments in nanotechnology in the last ten years, and it attracted considerable attention owing to their uniform nanoporosity, large surface area, outer-surface modification and in-pore functionality for tailoring the chemical properties of the material for anchoring specific guest moieties. MOF’s have been particularly highlighted for their excellent gas storage and separation properties. Recently biomolecules-based MOF’s were used as nanoencapsulators for antitumor and antiretroviral controlled drug delivery studies. However, usage of MOF material for removal of ionic detergent-SDS from biological samples has not been reported to date. Here, first time we demonstrate its novel applications in biological sample preparation for mass spectrometry analysis.

**Methods:**

SDS removal using MIL-101 was assessed for proteomic analysis by mass spectrometry. We analysed removal of SDS from 0.5 % SDS solution alone, BSA mixture and HMEC cells lysate protein mixture. The removal of SDS by MIL-101 was confirmed by MALDI-TOF-MS and LC-MS techniques.

**Results:**

In an initial demonstration, SDS has removed effectively from 0.5 % SDS solution by MIL-101via its binding attraction with SDS. Further, the experiment also confirmed that MIL-101 strongly removed the SDS from BSA and cell lysate mixtures.

**Conclusions:**

These results suggest that SDS removal by the MIL-101 method is a practical, simple and broad applicable in proteomic sample processing for MALDI-TOF-MS and LC-MS analysis.

## Background

Metals organic frameworks (MOF) are nano-porous compounds that contain metal ions or clusters that are connected by organic ligands to forms two or three dimensional structures. Last 10 years, MOFs are most potential compounds in hydrogen storage [1, 2], CO_2_ capture [3, 4], catalysis [5, 6], sensing [7], and others [8]. It has highly porous nature that makes very attractive for catalysis applications. In addition, MOFs have large diversity in nature as compared to Zeolites due to the use of SiO4/AlO4 tetrahedral building units in the later materials. The catalytic applications of zeolites are more restricted to relatively small organic molecules (typically no larger than xylene) because due to its microporous in nature. Whereas, the size of pore, shape, dimensionality and chemical natures in MOFs have been controlled by the suitable selection of their building blocks (metal and organic linker). Apart from these features, the lower acidity of the active centers in MOFs makes these materials even very attractive compared Zeolites (highly acidic centers). Also, MOFs may be changing the interactions of adsorbing reactants and the transition states or intermediaries formed inside the framework cavity between the host and guest.

The fundamental step of global proteomics experiments, particularly involving sensitive MS technique is efficient sample preparation. Extraction of total proteins from various biological sources including tissues and cultured cells using SDS-Sodium Dodecyl Sulfate, CHAPS, and Triton is well known in several biochemical studies. SDS is widely used and considered to be very beneficial due to complete cell lysis, disaggregation and efficient solubilization of the global proteome primarily hydrophobic membrane-bound components. SDS interacted with proteins by ionic and hydrophobic bonds and dissolves proteins by changing their secondary and tertiary structures [9]. Further, it plays an important role in studies of membrane proteins or aggregated proteins, because of these proteins are not soluble in other agents. In addition, SDS continuously used in protein separations from biological samples by sodium dodecyl sulfate- polyacrylamide gel electrophoresis (SDS-PAGE) method. Unfortunately, SDS in the samples can be unfavourable due to its unwanted effects in liquid chromatography and it produces large DS^−^ related signals and ion suppression effects [10, 11]. Therefore, SDS removal is an essential prerequisite for achieving higher peptide/protein coverage in the mass-spectrometric analysis. It can be accomplished by numerous techniques such as precipitation, strong cation exchange, protein and peptide level purification with pierce detergent removal cartridges and FASP II etc. [12]. Gu et al. 2011 [13] reported that the MOFs based material is very useful for biological applications. But, usage of MOFs material for removal of ionic detergent-SDS from biological samples has not been reported to date. Therefore, we tried to find out a novel method for removal of ionic detergent-SDS from biological samples by microdevices based MOFs material for proteomic analysis.

## Results and discussion

Standard sample cleans up with different solid phase extraction (SPE) or desalting methods have not been much effective in SDS depletion to date; prompting the investigation of various methods for SDS removal will make several commercial products [14], Filter aided sample preparation (FASP) [15], high salt precipitation kits [16], and SDS specific binding spin cartridges are very famous methods for depletion of detergent from protein mixtures for proteomic sample preparation. However, a majority of techniques are hindered by low protein recovery, labour intensiveness, irreproducibility and incomplete SDS removal. To these address, we tried to find out a novel and efficient method for SDS removal. Here we used MOFs (MIL-101) as a binding material for SDS removal from the biological samples by micro- device based method. Our preliminary results were highly encouraged and demonstrated a novel biological application of MOF materials which could have significant value in sample processing i.e SDS depletion for MALDI-TOF and LC-MS analysis.

### SDS removal from 0.5 % SDS solution using MIL-101

**Fig. 1:**
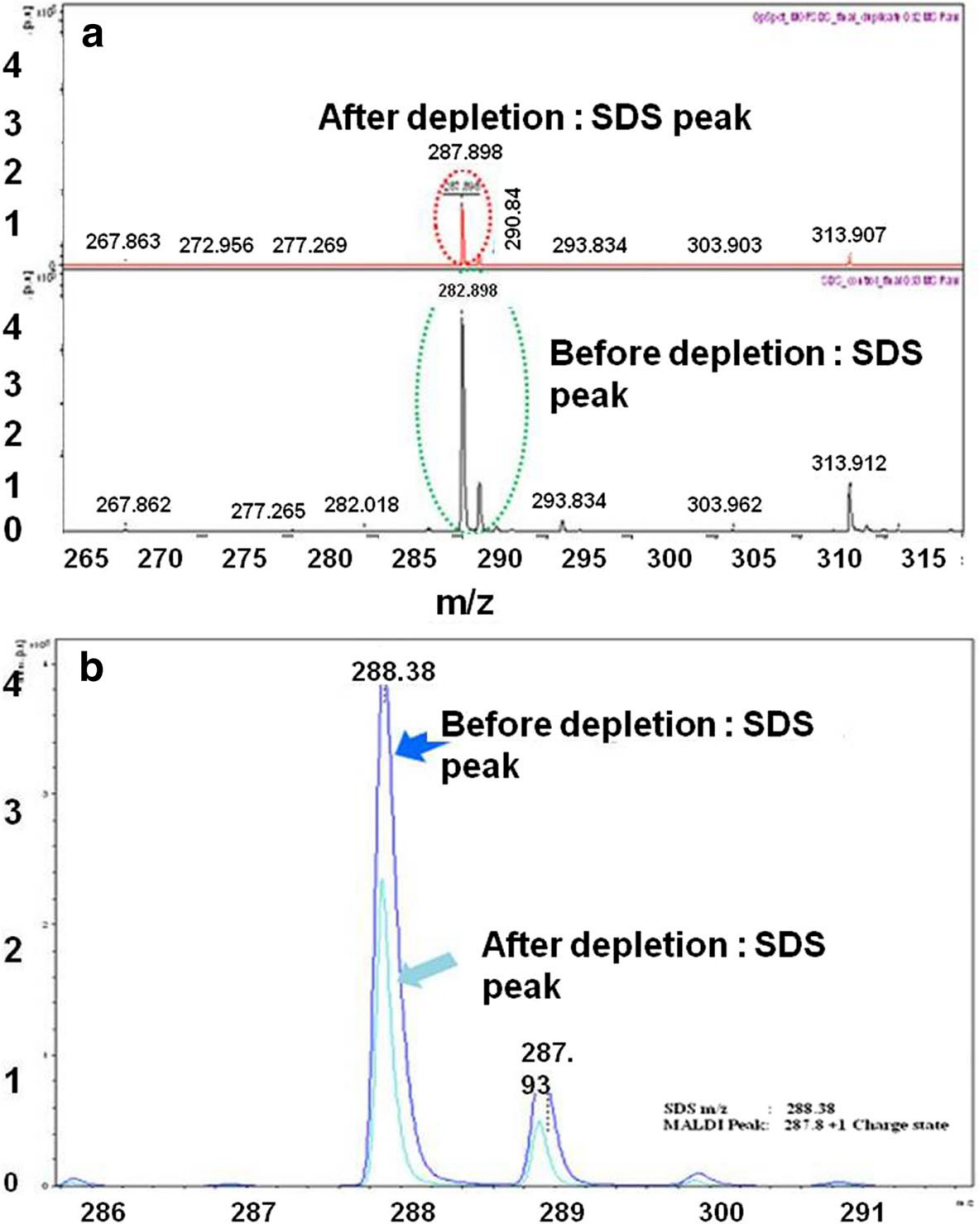
**a-b** SDS m/z intensity, before and After SDS removal by MIL-101, data acquired on MALDI-TOF MS

First, we tried to remove the SDS from 0.5 % SDS solution using MIL-101 as a binding material. Presently, this method has not been applied for removing SDS during proteomic analysis by the mass spectroscopy. Thus, we designed and used this method for SDS removable from biological protein mixture before MALDI-TOF-MS analysis. Initially, we removed the SDS from 0.5 % SDS solution with the help of MIL-101 material by centrifugation at 3000 rpm and the results exhibited that maximum concentration of SDS was removed as evidence the peak intensity of SDS at 287.89[M + H] was differed and clear in after removal of SDS (Fig. 1a & b). This result suggests that for the solution containing SDS can be removed by MIL-101 material.

### SDS removal from BSA mixtures using MIL-101

**Fig. 2:**
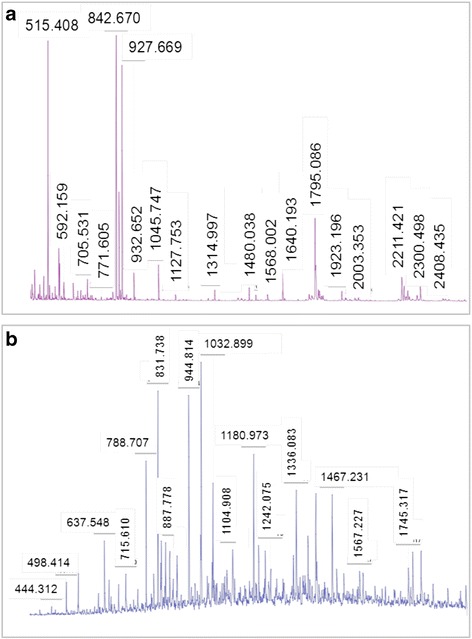
**a** PMF profile of SDS removed BSA tryptic digest mixture using MALDI-TOF. **b** PMF profile of SDS depleted tryptic digest mixture using MALDI-TOF

Further, we analysed capability of MIL-101 for removal of SDS from bovine serum albumin. Different concentration of BSA was mixed with 0.5 % of SDS. Then BSA in SDS mixture was treated with MIL-101 for overnight and then centrifuged at 3000 rpm prior to MALDI-TOF-MS analysis. Generally, SDS acts as a potent surfactant that could be denatured the trypsin activity when digestion process. But, we observed good signalling intensity for BSA tryptic peptides. It indicated that MIL-101 has an ability to remove the SDS from single protein mixture with robust trypsin activity (Fig. 2a).

### SDS removal from cell lysate protein mixture using MIL-101

Further, we planned to assess the weather MIL-101 have an ability to remove the SDS from cell lysate protein mixtures or not, because it is very complicated and little difficult to separate SDS from the biological samples as compared with single protein mixtures. So, we prepared protein extract from HMEC cells and mixed with 0.5 % boiling SDS. This mixture was incubated with 30 mg of MIL-101 slurry with end-over-end rotation overnight. Proteins were further separated from MIL-101 by centrifugation at 3000 rpm for 5 min. Supernatant containing protein mixture was processed for MALDI-TOF-MS analysis. This result suggests that MIL-101 has effectively removed SDS from cell lysate protein mixtures (Fig. 2b).

### LC-MS analysis of tryptic peptides

**Fig. 3:**
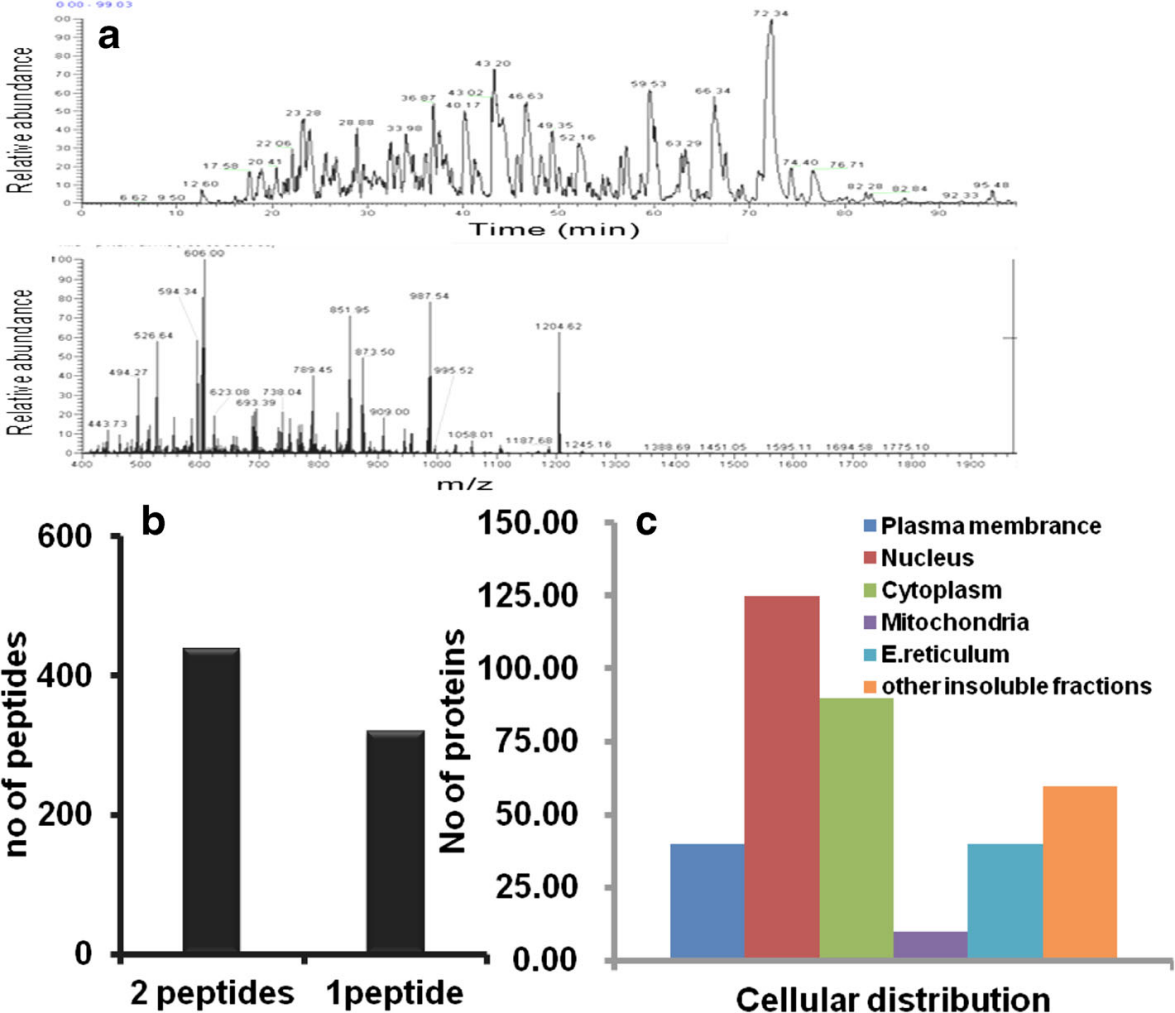
**a** Liquid Chromatogram separations of SDS removed tryptic. **b** Number of proteins with 1 or 2 unique peptide matches. **c** GO based annotations and cellular distribution

Finally, the processed sample was subjected to LC-MS analysis. The result indicates that MIL-101 processed sample shows little ion suppression effect only. It may be due to trace amount of SDS was found in the samples. Overall MIL-101 effectively removed SDS from cell lysate by its binding efficiency (Fig. 3a). Figure 3b demonstrated the number of proteins with 1 or 2 unique peptide matches, Totally 750 protein were identified; among them 438 proteins having two peptide matches and 319 proteins having one peptide hits. Figure 3c demonstrated the GO (Gene Ontology) based annotations and cellular distribution of identified proteins. From our preliminary analysis, we hypothesize that MIL-101 is an attractive MOFs candidate for SDS removal from biological proteins before MS analyses.

## Conclusions

The MIL-101 is an attractive MOFs candidate for removing SDS from biological samples through its electrostatic interactions. All the experimental results suggested that MIL-101 method could be a very useful method to prepare biological sample preparations for spectroscopy based proteomic analysis.

## Methods

### Chemical

BCA kit and HMEC cells were purchased from Thermo Fisher Scientific and American Type Culture Collection [Rockwille, MD, USA] respectively. SDS, Trypsin, BSA and DTT obtained from Sigma-Aldrich, USA. MIL-101 was from sigma product # 185361[Final concentration 100 μg/ml] and Microdevices obtained from Mobitec; product code: MobiSpin Column F (1.5 ml tubes).

### SDS removal from 0.5 % SDS solution

MIL-101 (100 μg slurry) was mixed with 0.5 % SDS solution and incubated at room temperature for overnight. Then, the sample was centrifuged at 3000 rpm for 5 min and then subjected to MALDI-TOF analysis.

### SDS removal from BSA mixture

Different concentrations of BSA (5, 10, 25 μg) were mixed with the freshly prepared SDS (0.5 % SDS w/v) and was further incubated overnight with the slurry prepared using 100 μg MIL-101 with one ml of freshly prepared ice cold phosphate buffer and reduced with 10 mM DTT. Later, the sample was subjected to in-solution digestion using trypsin and peptides were desalted and then loaded into MALDI-TOF analysis.

### SDS removal from cell lysate mixture

**Fig. 4:**
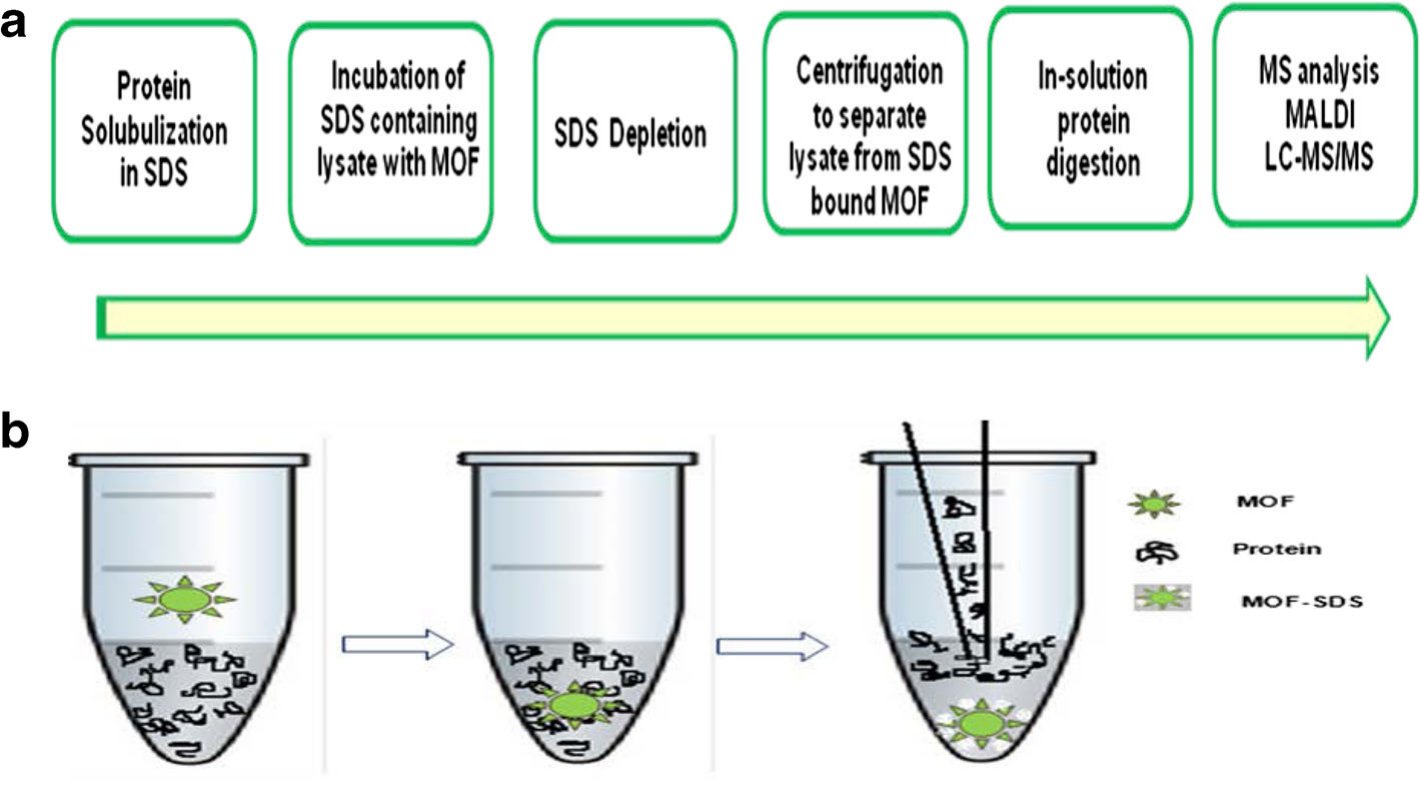
**a** Work flow of SDS removal from a complex lysate mixture. Microdevices based **b** SDS removal using MIL-101, SDS was bound to MOF and proteins were separated by Centrifugation for downstream analysis

HMEC cells were lysed using 0.5 % boiling SDS and sonicated to clear the chromatin. Lysate protein was estimated by BCA method and incubated with MIL-101 matrix (100 μg slurry) at room temperature for overnight to SDS attraction by the MOF and then separated by centrifugation. Protein mixture was in-solution digested, desalted and then subjected into MALDI-TOF and LCMS analysis. An outline of the work flow was illustrated in Fig. 4a-b.

### MALDI-TOF analysis

Peptide mass fingerprinting (PMF) was conducted using MALDI-TOF-MS equipped with linear mode 20 KvA, laser shots 150 (337 nm, 50 H, N2 laser (Bruker Daltonics, Germany). For, each sample spectra required in the positive linear mode, and an average of 200 spectra that passed the accepted criteria of peak intensity was automatically selected and accumulated. Spectrum processing and data base searches were conducted b automatic mode with internal calibration using trypsin autolysis peaks (m/z 842.509 and m/z 2211.104). The fragmentation of selected peptide was measured using the PSD mode for MS analysis [17].

### LC-MS analysis

The peptides were analysed using a reverse phase capillary column (LC system -LTQ; Thermo Scientific, San Jose, CA) prepared by slurry packing 3-μM Jupiter C18 bonded particles into a 65 cm long and 75 μM inner diameter fused silica capillary. 2.5 μg of peptides were loaded onto the column; the mobile phase was held at 100 % buffer A (0.1 % formic acid) for 20 min, followed by a linear gradient from 0 to 70 % buffer B(0.1 % formic acid in 90 % acetonitrile) for more than 85 min. Each full MS scan (m/z 400–2000) was followed by collision induced MS/MS spectra. The dynamic exclusion duration was set to 1 min; the heated capillary was maintained at 200 °C and the ESI voltage was held at 2.2 kV [18].

## Abbreviations

SDS: Sodium dodecyl sulfate
MOFs: Metal organic frameworks
HMEC: Human mammary epithelial cells
MALDI-TOF: Matrix assisted laser desorption/ionization-time of flight
BSA: Bovine serum albumin
DTT: Dithiothreitol
GO: Gene ontology
LC-MS: Liquid chromatography- mass spectrometry
FASP: Filter aided sample preparation)
SPE: Solid phase extraction
CHAPS: 3-[(3-cholamidopropyl)dimethylammonio]-1-propanesulfonate
